# Metrology with a twist: probing and sensing with vortex light

**DOI:** 10.1038/s41377-024-01665-1

**Published:** 2025-01-01

**Authors:** Mingjian Cheng, Wenjie Jiang, Lixin Guo, Jiangting Li, Andrew Forbes

**Affiliations:** 1 School of Physics, Xidian University, South Taibai Road 2, Xi’an, 710071 Shannxi China; 2 School of Physics, University of the Witwatersrand, Private Bag 3, Johannesburg, 2050 South Africa

**Keywords:** Optical metrology, Atmospheric optics

## Abstract

Optical metrology is a well-established subject, dating back to early interferometry techniques utilizing light’s linear momentum through fringes. In recent years, significant interest has arisen in using vortex light with orbital angular momentum (OAM), where the phase twists around a singular vortex in space or time. This has expanded metrology’s boundaries to encompass highly sensitive chiral interactions between light and matter, three-dimensional motion detection via linear and rotational Doppler effects, and modal approaches surpassing the resolution limit for improved profiling and quantification. The intricate structure of vortex light, combined with the integration of artificial intelligence into optical metrology, unlocks new paradigms for expanding measurement frameworks through additional degrees of freedom, offering the potential for more efficient and accurate sensing and metrological advancements. This review aims to provide a comprehensive overview of recent advances and future trends in optical metrology with structured light, specifically focusing on how utilizing vortex beams has revolutionized metrology and remote sensing, transitioning from classical to quantum approaches.

## Abstract (web summary)

AI algorithms analyzes the OAM spectrum and intensity patterns of twisted light as it propagates through a turbulent medium, enabling the detection of the key features of the medium.

## Graphical abstract

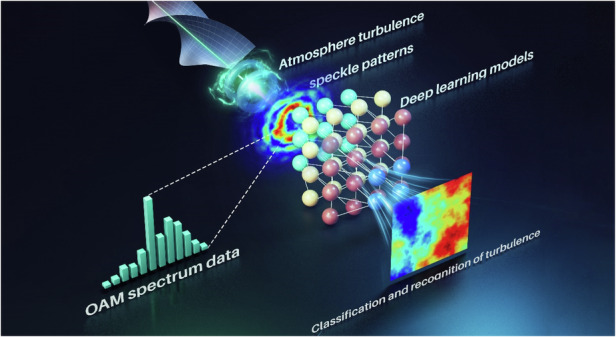

## Introduction

Vortex beams represent a significant milestone in modern optical science, driven by profound scientific discoveries and rapid technological advancements^1^. Distinguished by their unique helical phase front and the revolutionary concept of orbital angular momentum (OAM), vortex beams have ushered in a new era in the understanding and utilization of laser technology, expanding the boundaries of what was once thought impossible^2^. Since the seminal contributions by Allen et al. in 1992^3^, which connected OAM to the spatial structure of vortex beams, these beams have captured the imagination of the scientific community. Their distinctive spatial structures have sparked a revolution leading to remarkable advancements in optics and quantum mechanics, thereby driving continuous technological progress^4,5^.

**Fig. 1:**
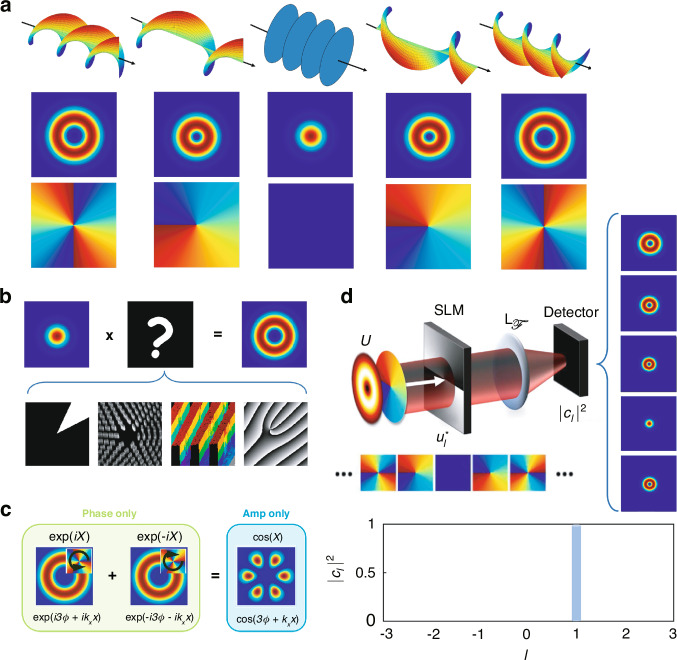
Creation and detection of vortex beams. **a** OAM beams are defined by their twisted wavefronts and azimuthal phase, giving rise to donut-shaped intensity patterns, illustrated here for topological charges from 0 to ±2, the sign determining the direction of the phase change. **b** Some examples of optical transformations for tailoring OAM beams from an input Gaussian beam, shown from left to right as a pie-shaped amplitude mask, a meta surface, a diffractive optic, and a digital hologram of a forked grating. **c** Phases and amplitudes can be swapped by noting that two phase-only functions can produce an amplitude-only function, shown here for the example *X* = 3*ϕ* + *k*_*x*_*x*. **d** The detection of OAM is possible by running the creation in reverse for modal decomposition, requiring a hologram and an on-axis measurement in the far-field with the aid of a Fourier transforming lens, i.e., at (*k*_*x*_, *k*_*y*_) = (0, 0). An input OAM mode is untwisted back to a Gaussian when the SLM has the complex conjugate of the incoming mode, returning a modal spectrum (∣*c*_*l*_∣^2^) of the initial beam, shown here for an *l* = 1 input mode

It is well established that photons can carry spin angular momentum (SAM) of ±*ℏ* through circularly polarized light, with the direction determined by its handedness (left- or right-circularly polarized). In 1992, it was demonstrated that the OAM of vortex light is intrinsically linked to its spatial phase structure. Specifically, light with an azimuthal phase described by $\exp (-{\rm{i}}l\phi )$, where *l* denotes the topological charge and $\phi =\arctan (y/x)$ is the azimuthal angle, imparts an OAM of *lℏ* per photon in the *z*-direction^3^. This phase endows the light beam with a helical wavefront structure along the propagation direction, twisting ever tighter as the OAM increases, as shown in Fig. 1a, where the twisted nature of the wavefront and phase gives rise to donut-shaped intensity patterns. The unique properties of vortex light, such as its ability to exert torque on particles and influence their rotational motion, directly arise from this helical phase structure. Additionally, the phase singularity at the core, where the intensity is zero, results in an undefined azimuthal phase, leading to the formation of optical vortices, regions of null intensity.

Angular momentum is not restricted to the propagation direction; it can also manifest as transverse components. These components appear as fields with transverse SAM, termed photonic wheels due to their transversely spinning electric fields^6^, or as fields with transverse OAM, which exhibit phase twisting in the (*y*, *z*) plane rather than the (*x*, *y*) plane^7^. Although OAM beams are often referred to as vortex beams with a topological charge *l*, a beam can have zero OAM while possessing a nonzero total topological charge, and conversely, a beam can have nonzero OAM while having a zero total topological charge^8^. SAM and OAM can be combined in a vectorial superposition to form vector vortex beams. For example, combining opposite spin states with opposite OAM helicities, such as $\left\vert +\sigma \right\rangle \left\vert +l\right\rangle +\left\vert -\sigma \right\rangle \left\vert -l\right\rangle$, results in what is typically referred to as cylindrical vector vortex beams^9^.

Such OAM fields can be precisely tailored using various techniques, including amplitude control^10^, dynamic phase modulation^11^, geometric phase manipulation^12^, complete phase control^13^, and direct laser production^14^, each involving different optical elements, as detailed in Fig. 1b. Among these techniques, spatial light modulators (SLMs) are particularly valuable for dynamically adjusting both the amplitude and phase of light beams, thereby enabling the creation of complex field patterns. It is also possible to interchange phase and amplitude. For instance, as shown in Fig. 1c, two phase-only functions of opposite sign, $\exp (\pm iX)$, can be added to produce an amplitude-only function, $\cos (X)$. In the context of OAM, this means adding two OAM modes of opposite topological charge, as shown in the example *X* = ±3*ϕ*. The extra ±*k*_*x*_*x* term is a grating function, often used to move the desired mode off-axis.

Detection methods, particularly relevant to this review, can employ projective techniques such as the modal decomposition method^15^. This approach decomposes the light field into its constituent modes, providing a detailed understanding of the field’s structure and dynamics. The decomposition is described by the expression:

1 $$U(\phi )=\sum {c}_{l}\exp ({\rm{i}}l\phi )$$

Here, *U*(*ϕ*) represents the light field as a function of the angular coordinate *ϕ*, with the summation over the mode indices *l*. The coefficients *c*_*l*_ quantify each mode’s contribution to the overall field and are determined by the orthogonality condition of the modes. They are computed using the integral:

2 $${c}_{l}=\langle U| \exp ({\rm{i}}l\phi )\rangle =\mathop{\int}\nolimits_{\!0}^{2\pi }U(\phi )\exp (-{\rm{i}}l\phi )d\phi$$

This integral projects the light field onto the basis functions $\exp ({\rm{i}}l\phi )$, with the coefficients *c*_*l*_, which are complex numbers, reflecting both the amplitude and phase information of the corresponding mode.

To ensure the decomposition accurately represents the light field, the coefficients must satisfy a normalization condition:

3 $$\sum | {c}_{l}{| }^{2}=1$$

This condition guarantees that the total power of the light field is conserved and properly distributed among the various modes.

Modal decomposition allows for the analysis and interpretation of complex light fields in terms of simpler, orthogonal modes. This method is particularly useful in optical communications for more efficient encoding and transmission of information. Additionally, it has applications in optical metrology and imaging, where precise characterization of the light field is crucial for accurate measurements and high-resolution imaging.

The typical experimental scheme for measuring the OAM spectrum is as follows: an optical field *U* is modulated by a hologram of ${u}_{l}^{* }=\exp (-{\rm{i}}l\phi )$, which is encoded onto a rewritable medium using SLMs or digital micro-mirror devices. This field is then Fourier transformed using a Fourier lens ${L}_{{\mathcal{F}}}$. It can be shown that the on-axis intensity in Fourier space is proportional to the inner product $| {c}_{l}{| }^{2}={\left\vert \langle U| \exp ({\rm{i}}l\phi )\rangle \right\vert }^{2}$. The corresponding normalized modal distribution, commonly referred to as the spiral spectrum, is subsequently extracted from this intensity in Fourier space (examples for $l=[-3,3]\in {\mathbb{N}}$ are depicted in Fig. 1d), demonstrating its similarity to a frequency spectrum^16^. Alternative detection methods include deterministic sorting of OAM modes based on conformal mapping^17^ and interference^18^.

The emergence of vortex beams has catalyzed innovations across a wide spectrum of applications, including optical communications, microscopy, micromanipulation, and beyond^19–22^. In optical communications^23^, vortex beams have revolutionized modulation, encoding, and multiplexing techniques, enhancing data transmission security through their unique OAM states. Additionally, their interaction with matter in optical microscopy has facilitated enhanced profiling and quantification, providing unprecedented precision and resolution that surpass the capabilities of traditional microscopy techniques^24,25^.

Historically, optical metrology has relied on linear phase gradients formed by the interference of two plane waves resulting in intensity fluctuation patterns known as fringes. While foundational, this traditional approach has its limitations. By leveraging a broader spectrum of light’s physical properties and pioneering innovative techniques, researchers have achieved significant advancements in optical metrology. A key development in this context is the introduction of vortex beams, representing a major breakthrough and positioning them as a superior choice for advanced optical metrology methods. These beams, with their unique phase structure and OAM properties, offer substantial improvements in measurement accuracy and resolution over traditional light sources^26^. The transformative nature of vortex beams not only enhances overall measurement capabilities but also expands the potential applications of optical metrology to unprecedented levels.

**Fig. 2:**
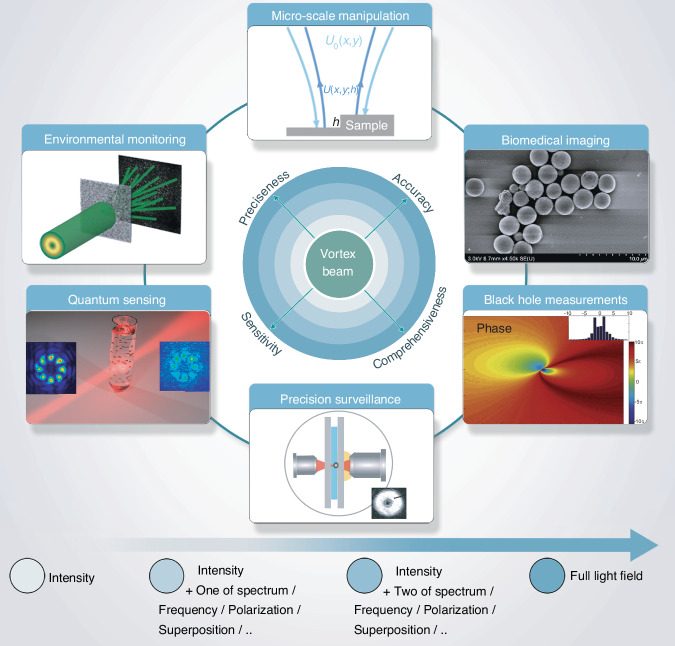
Evolution of optical metrology capabilities. This progression highlights the transition from utilizing basic intensity attributes to leveraging a broader spectrum of light’s physical properties, ultimately leading to the full exploitation of available data. A significant driver of this evolution is the application of vortex beams, which possess unique phase structures and OAM properties, which enhance measurement precision and resolution. Vortex beams facilitate precise micro-scale engineering, biomedical research such as advanced imaging and probing, and deep-space exploration, including improved black hole measurements through OAM, increased precision surveillance, quantum sensing, and environmental monitoring. This ongoing journey toward fully utilizing light’s physical properties promises unprecedented improvements in optical metrology’s precision, accuracy, comprehensiveness, and sensitivity

One of the major advantages of incorporating vortex beams in metrology is their ability to measure three-dimensional (3D) motion, encompassing both rotational and translational velocities of moving objects, through linear and rotational Doppler effects^27^. The integration of vortex beams into optical metrology signifies a major advancement in measurement and analytical capabilities. This progress has had a profound impact on various scientific and technological fields requiring exceptional precision and detailed analysis^28,29^. Notable areas benefiting from these innovations include micro-scale engineering, biomedical research, deep-space exploration, precision surveillance, quantum sensing, and environmental monitoring. Figure 2 highlights a critical future direction in optical metrology by illustrating the integration of additional light properties or degrees of freedom. This evolution begins with the application of one-dimensional intensity attributes of vortex beams, advances to two-dimensional (2D) attributes combining intensity with another physical property, and progresses to 3D attributes incorporating intensity along with two additional physical properties. Ultimately, utilizing the full range of available physical properties will significantly enhance the precision, accuracy, comprehensiveness, and sensitivity of optical measurements.

Vortex beams, which have transitioned from theoretical constructs to vital elements in modern optics, offer considerable potential for future advancements. Progress in optical processing and manipulation technologies has significantly enhanced the ability to harness the unique OAM properties of vortex beams. These beams surpass other types in their capacity to utilize various physical attributes of light beams, leading to groundbreaking discoveries and novel applications^30^. This paper presents compelling examples and case studies that demonstrate the significant potential of vortex beams in various metrology applications. These cases underscore how vortex light enables high-precision measurements, improves resolution, and expands existing metrology techniques. The primary objective is to highlight that the intricate structure of vortex light conveys a wealth of information that offers great potential for the advancement of metrology.

## Detecting dynamics with vortex light via the rotational Doppler effect

The conventional methodology for tracking system dynamics with light relies on the Doppler effect, widely utilized to measure the velocities of objects or fluids via frequency shifts. However, the standard linear Doppler effect is inherently limited since it can only capture the movements towards or away from the observer, leaving lateral and rotational movements undetected. To overcome this limitation, it is essential to delve into the spatial structure of light^31^. In scalar vortex beams, the Poynting vector features an azimuthal component at each position across the beam, indicating the flow of optical momentum in a circular path around the beam’s axis. The azimuthal phase structure of probe beams, upon interaction with the rotational dynamics of either the observer or the illuminated object, provides valuable information about the object’s position and velocity, resulting in a frequency shift related to transverse position changes, termed the rotational Doppler effect (RDE)^32^. This shift is directly proportional to both the rotational angular velocity and the OAM mode number^29^^,^^33,34^, owing to the azimuthal variation of the vortex beam’s phase at a rate of *l* × 2*π* per circle, which interacts with the rotational motion to produce the frequency shift. Consequently, vortex beams, when scattered or reflected by a rotating object, produce a rotational Doppler frequency shift that can be multiple times larger than that induced by circularly polarized beams. Both theoretical analysis and experimental verification have confirmed the effectiveness of vortex beams in measuring rotational velocities^35^.

**Fig. 3:**
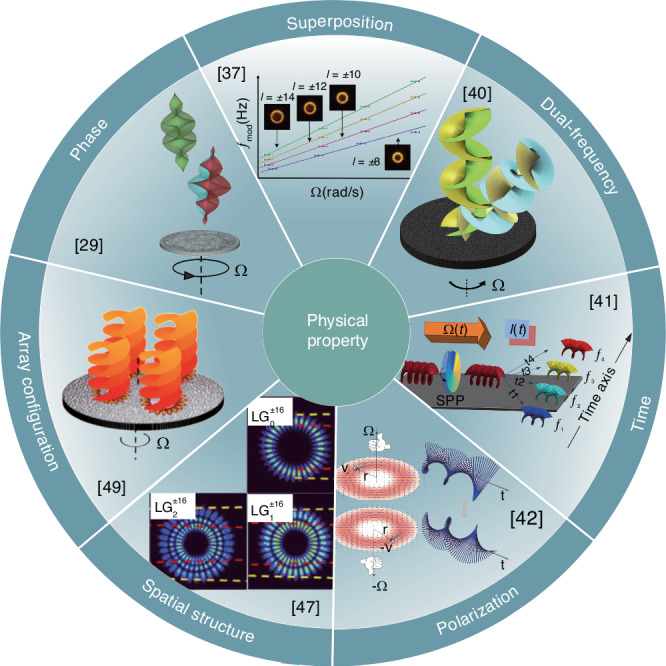
OAM Doppler metrology. Enhancing Doppler metrology with a helical phase of vortex beams, by integrating superposition, dual-frequency, time, polarization, spatial structures, and array configurations, improves signal amplitude, accuracy, and robustness while enabling rotational direction and acceleration analysis. Amplified RDE arises when opposite OAM modes interact with a rotating object. Dual-frequency vortex beams elevate Doppler signals to higher frequencies, reducing noise and enhancing rotational direction analysis. Time-frequency analysis allows dynamic monitoring of angular velocity and acceleration, while polarization and OAM within a vector RDE framework support simultaneous evaluation of velocity and direction. Exploring the spatial structure of probing beams enhances detection accuracy and efficiency for complex radial targets, and an array of superimposed optical vortex beams increases the signal amplitude and robustness against non-coaxial incidence. References given in the insets

The integration of helical phase elements into Doppler metrology, facilitated by vortex beams, significantly enhances its capabilities, particularly when enriched with multiple degrees of freedom, such as superposition, dual-frequency, time, polarization, spatial structures, and array configurations, as depicted in Fig. 3. Unlike the conventional linear Doppler effect, which pertains to the linear momentum of the incident beam, the RDE maintains the OAM of the incident beam unchanged^36^. The RDE becomes evident when a superposition of two distinct OAM modes, *l*_1_ and *l*_2_, interacts with a rotating object, resulting in an amplification of the rotational frequency shift that is proportional to the absolute difference ∣*l*_1_ − *l*_2_∣^37,38^. RDE detection relies on an internally correlated OAM spectrum rather than the coherence of the single incident mode^39^, enabling its application even with incoherent sources. This sophisticated approach is further exemplified by using dual-frequency vortex beams as probes, which transmute Doppler signals from the low-frequency domain to the high-frequency realm, thus minimizing noise and enhancing rotational direction analysis^40^. Time-frequency analysis enables the dynamic monitoring of angular velocity changes over time, providing an evaluation of rotational motion variations^41^. Integrating multiple light properties in a kilometer-scale air-core optical fiber velocimeter has demonstrated the measurement and analysis of vector angular velocities by combining polarization and OAM within a vector RDE framework^42,43^. This method facilitates the simultaneous assessment of velocity and direction in smaller objects through the interference of oppositely polarized vortical beams^44^ and exploits inhomogeneous polarization patterns for superior motion detection^45,46^. Exploring the spatial structure of probing beams enhances the accuracy and efficiency of RDE detection, particularly for targets with complex radial structures, by leveraging the radial and angular modes of LG beams^47,48^. Additionally, employing an array structure of superimposed optical vortex arrays demonstrates enhanced signal amplitude and robustness against non-coaxial incidence through the holistic OAM’s modal gathering effect^49^.

**Fig. 4:**
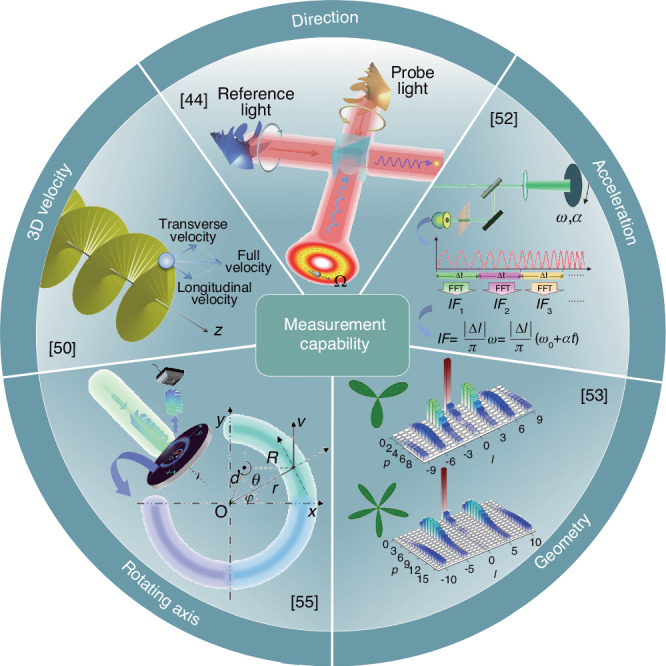
Advances in Doppler metrology. Doppler metrology technology has evolved from assessing the 2D rotational velocity to covering 3D velocity assessment, determination of rotational direction and acceleration, geometric symmetry analysis, and detection of axis orientation. 3D velocity trajectories of particles can be analyzed by revealing both translational and rotational velocities, including helical motions. The rotational axis and direction can be determined by using dynamic structured light, dual-frequency vortex beam, or vector RDE with oppositely polarized vortex beams. Time-frequency analysis dynamically tracks rotational velocity and obtains acceleration. RDE measurements exhibit high sensitivity to geometric symmetry and rotation axes of objects, enabling analysis of geometric symmetry and determination of rotation axes. References given in the insets

Optical metrology technology employing the RDE of vortex beam has advanced significantly, evolving from assessing 2D dynamics to conducting comprehensive 3D evaluations. Initially focused on rotational velocity, this technology now encompasses the determination of rotational direction, measurement of acceleration, geometric symmetry analysis, and axis orientation detection, as demonstrated in Fig. 4. This progression is exemplified in the utilization of LG beam illumination to reveal both translational and rotational velocity components of particles on 3D helical trajectories^50^. Distinct frequency shifts arising from the rotational and longitudinal motions of these particles facilitate the determination of their movement direction. Specifically, aligning the probe’s phase rotation with the particle’s rotation minimizes the frequency offset, whereas opposing alignments increase it. This variation in frequency, induced by dynamic structured light, aids in analyzing movement direction^51^. Alternatively, the rotational direction can also be determined using dual-frequency vortex beams as probes^40^ or by analyzing the interference of oppositely polarized vortex beams^44^.

Time-frequency analysis, which dynamically tracks rotational velocity^41^, is further enhanced by integrating OAM modulation. This integration enables the simultaneous assessment of both rotational velocity and acceleration, thus improving measurement precision without adding complexity^52^. The application of rotational Doppler frequency shift as a diagnostic tool has not only enabled the analysis of rotating velocity and geometric symmetry over extensive outdoor distances^53^ but has also been extended to infrared light detection through second harmonic generation, exemplified by up-conversion detection of RDE frequency^54^. The spliced superimposed vortices with asymmetric defects exhibit high sensitivity to the rotation axes of objects, utilizing them as a probing beam can rapidly determine the rotation axes, which is essential for the alignment of the rotating axis of the rotating surface^55^.

**Fig. 5:**
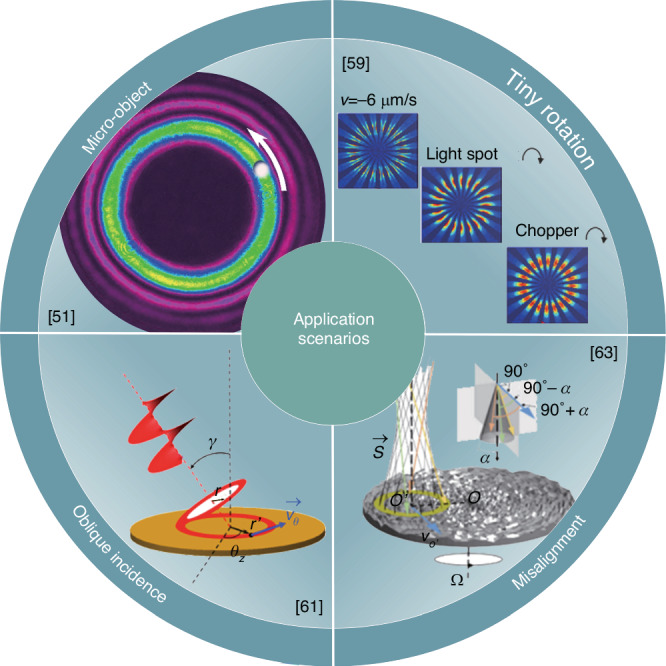
Applications of the rotational Doppler effect (RDE) metrology. RDE metrology with vortex beams exhibits exceptional adaptability across various conditions, ranging from ideal to complex scenarios such as micro-object rotation detection, tiny rotations, oblique incidence, and lateral misalignment. Utilizing vortex beams with opposite-sign OAM in both reference and measurement paths, this approach accurately identifies micro displacements, the direction of micro-object rotation, and minute velocity changes. The central frequency remains stable despite variations in incident angle and lateral displacement, while the rotational Doppler signal broadens, ensuring extraction of rotational velocity even in cases of oblique incidence and lateral misalignment. References given in the insets

Theoretical and experimental research has significantly advanced RDE metrology, enhancing its adaptability across a range of complex application scenarios, from ideal incident conditions to challenging scenarios such as micro-object rotation detection, tiny rotations, oblique incidence, and lateral misalignment, as presented in Fig. 5. The superposition interference of two different OAM modes at the detector amplifies the observed Doppler shift, allowing for detailed examination of microorganism motility^56,57^. Monitoring the rotation angle and direction of the interference petal, generated by coaxial interference between the measured and reference vortex beams, enables the detection of micro displacement and movement direction^58^. By employing two vortex beams with opposite-sign OAM in the reference and measurement paths, an interference pattern can discern tiny changes in velocity through the rotation of petal-shaped light spots^59,60^, whose velocity correlates directly with the time-varying phase difference.

Traditionally, RDE techniques require aligning the beam axis with the object’s rotation axis, limiting practical applications, especially for non-cooperative targets. However, advancements have facilitated the accurate extraction of rotational velocity even under oblique incidence by exploiting the central frequency, which remains invariant to the incident angle^61^. Moreover, in laterally misaligned systems, the central rotational Doppler frequency shift remains constant, while the rotational Doppler signal broadens in the spectrum, leading to proportional expansion of the signal bandwidth relative to the lateral displacement distance, thereby enabling extraction of valuable information regarding the rotating object^62,63^. Under non-coaxial incidence, the frequency spectrum broadens, and lateral displacements and angular deflections modify the distribution of OAM modes^64^. By utilizing the frequency difference between two adjacent RDE signals, the rotational frequency is determined, unaffected by the orientation of the measured object and the topological charge of the probe beam^65,66^.

When concerning the detection of complex rotating bodies and motion states, such as rough surfaces^67,68^, radially periodic moving surface^69^, rotator^70^, ideal rotating propeller^71^, and circular procession^72^, RDE measurements must be adaptable to increasingly complex sensing and metrology scenarios. Various probing beams, such as Bessel–Gauss beams^73^, perfect vortex beams^74,75^, ring Airy Gaussian vortex beams^76^, and combined vortex beams^77^, and fragmental optical vortex^78^ have been investigated to enhance the robustness and precision of RDE measurements. These alternative probing beams have demonstrated promising results, including improved signal-to-noise ratio, increased accuracy in measuring rotational velocity, and suitability for scenarios involving obstacles or long distances. However, it is worth noting that all of the aforementioned beams have been designed as 2D transverse optical fields. There is a wealth of multi-dimensional light field information available for exploration and analysis, with the potential to improve the performance of Doppler metrology technology. An intriguing yet unexplored possibility arises from utilizing 3D structured light as an illumination source to conduct detailed studies of motion across all degrees of freedom. Additionally, there is an enticing opportunity to explore the capabilities of 4D structured light, encompassing both spatial and temporal dimensions, for achieving ultrafast dynamics detection.

## Profiling complex structures with the OAM spectrum of optical vortex

In addition to the rotational Doppler frequency shift observed in vortex beams, their OAM spectrum can be directly exploited, serving as a valuable probe for investigating objects with complex structures. This capability provides an additional degree of freedom and conserved quantities for evaluating optical and mechanical systems. Since OAM is conserved in a closed system, any changes in the OAM spectrum of the probe beam can reveal valuable information about the interacting system. Utilizing the OAM or spiral spectrum of vortex light has revolutionized the acquisition of information about distant targets^79^, offering significant improvements over conventional methodologies^80^. By illuminating complex objects with vortex beams and analyzing kinematic data from the OAM spectrum, researchers can better assess the complex structures and dynamic behaviors of these objects, a challenge often faced with conventional optical acquisition techniques.

**Fig. 6:**
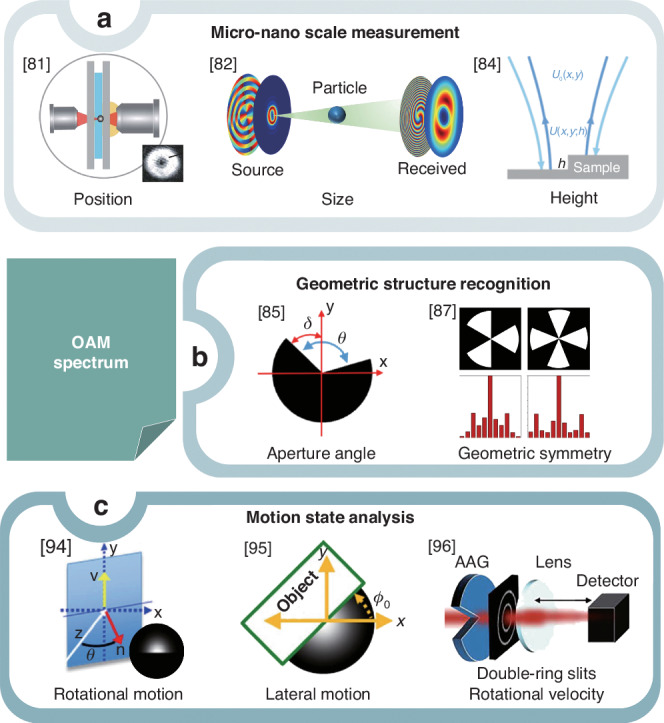
Utilizing the OAM spectrum of probe beams for complex object assessment. **a** The distinct OAM spectrum provides an additional degree of freedom and conserved quantities, facilitating high-precision measurements at micro- and nano-scale levels. Vortex light serves as an exceptionally sensitive probe. **b** Leveraging the OAM spectrum allows for rapid and accurate identification of geometric structures by correlating the positions of intensity minima to the object’s aperture angle and the gradient of the OAM phase spectrum to the object’s angular orientation. **c** Asymmetry in the OAM spectrum, induced by a moving obstructing object, directly reveals the object’s rotational and lateral motions, with phase differences in the OAM spectrum providing the determination of its rotational dynamics. References given in the insets

An in-depth analysis of the discrete OAM spectrum, derived from the probing beams interacting with complex objects, provides comprehensive insights into various attributes of these objects. This analysis facilitates precise measurements at the micro-nano scale and enhances the recognition of geometric structures and the analysis of motion states, as illustrated in Fig. 6. In optical metrology, vortex light serves as an exceptionally sensitive probe, with its OAM spectrum providing essential information for high-precision measurements at the micro- and nanoscales (Fig. 6a). The distinct OAM spectrum, obtained from the scattering or reflection of a probe beam by spherical particles with varying parameters, provides a crucial theoretical foundation for extracting important information about the dielectric spheres at the micron scale, including their size, position, ellipticity, and the refractive index of the surrounding medium^81^, particularly when enhanced by deep learning-based inversion techniques^82^. Vortex beams have also been instrumental in enhancing measurement sensitivity^83^, particularly for sub-nanometer step height measurements, enabling reliable and real-time assessments through fully digitized techniques without the need for reference or calibration measurements^84^.

The OAM spectrum analysis is essential for identifying complex geometric structures. Specifically, the positions of dips in the OAM intensity spectrum are directly correlated with the object’s aperture angle, while the gradient of the OAM phase spectrum is influenced by the object’s angular orientation. This relationship enables the simultaneous measurement of both the aperture angle and orientation of geometric objects^85^ (Fig. 6b). By integrating machine learning into this process, the methodology is further optimized, facilitating efficient target recognition with minimal computational cost. The aperture angle and orientation can be accurately determined solely from the intensity patterns of the received vortex beams^86^. Digital spiral imaging leveraging the OAM spectrum enables rapid and precise geometric structure recognition

The application of the OAM spectrum in digital spiral imaging experiments has successfully enabled the rapid and precise identification of the geometric symmetry of objects^87^. Digital spiral imaging posits that the OAM spectrum can reconstruct any image, and incorporating a time-dependent phase factor into each OAM mode allows for manipulations like image rotation and inversion^88^. In comparison to Fourier transforms, the LG mode spectrum maintains stability even during high-speed rotation, which is highly valuable for tracking rapidly rotating objects^89^. The combination of the RDE technique of vortex light with OAM spectrum analysis has demonstrated remarkable success in detecting and characterizing the geometric symmetry of rotational patterns^90,91^. The utilization of spatially and temporally controlled asymmetric perfect vortex basis has enabled diverse pulse train design, thereby contributing to effectively identifying amplitude targets and accurately sensing wavefront phases^92^. Additionally, the expansion of OAM scanning has improved azimuth resolution, thereby enabling finer precision across a wider range and facilitating accurate target measurements leveraging OAM spectrum-azimuth duality^93^. The OAM spectrum as a probe provides a robust toolkit for applications involving intricate objects or rotational movements in challenging application scenarios. When integrated with additional dimensions of data from the probe beam, its performance can be enhanced even further.

Furthermore, the asymmetry in the OAM spectrum, caused by an obstructing moving object, acts as a direct indicator of the object’s lateral motion, thus eliminating the need for image reconstruction and relying solely on a single light beam for motion detection^94^ (Fig. 6c). The phase differences in the OAM spectrum of the probe beam are directly proportional to the object’s rotational orientation, enabling the determination of rotational motion through these phase differences, analogous to Stokes polarimetry^95^. Moreover, the coherence-OAM matrix, recorded by a single-pixel detector, streamlines the assessment of angular shifts or rotational velocity in azimuthally symmetric objects, providing in-depth insights into the directional characteristics of the beams or interacting objects^96^.

An example at galactic scale is the detection of black holes, traditionally exploiting their effects on nearby matter and light. This includes studying changes in the direction and phase of emitted light, which can provide valuable insights into the nature of these enigmatic cosmic entities. Incorporating OAM into observational techniques has significantly expanded our capabilities in studying celestial objects, particularly those situated near black holes’ event horizons^97^. Vortex beams, characterized by their dark cores, play a crucial role in enhancing weak background signals and enabling precise astronomical measurements^98^. For instance, the use of an optical vortex coronagraph and adaptive optics led to the successful suppression of the primary star of a binary system by 97%^99^. Building upon this success, the Asiago 122 cm telescope employed an optical vortex coronagraph to generate and study an *l* = 1 optical vortex using starlight beams^100^.

**Fig. 7:**
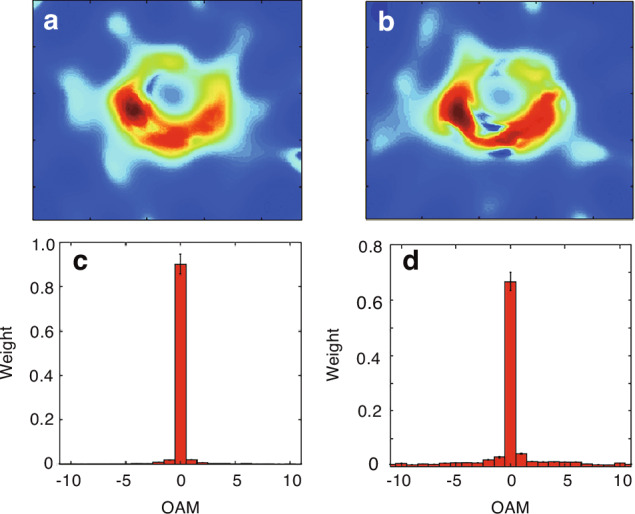
Observation of twisted waves from the black hole Einstein ring. **a**, **b** Normalized magnitudes of the electric field component along the observer’s direction, reconstructed through the transport of intensity equation analysis of the brightness temperature within a finite frequency bandwidth for epoch 1 and epoch 2. **c**, **d** The corresponding OAM spectrum. Adapted with permission from ref. ^103^

The use of multi-point interferometers has significantly advanced astrophysics by enabling the probing of the OAM of optical vortices in electromagnetic waves from astronomical objects, paving the way for new explorations of black holes^101^. By precisely measuring phase and wavefront distortions caused by black holes, scientists can now predict and confirm the presence of rotating black holes through direct observations of their OAM spectrum^102^. These advances in observational techniques enable more sophisticated measurements, validation, and further exploration of rotating black holes. For instance, both the OAM and electromagnetic vorticity were reconstructed using public images displaying the brightness temperature of the Einstein ring encircling the M87 black hole^103^, as shown in Fig. 7. Harnessing the additional information provided by OAM not only helps in determining a compact object’s true rotation but also allows scientists to use rotating black holes as laboratories for examining the principles of General Relativity^104^. Note that since these experimental observations are conducted at millimeter wavelengths, the effects of turbulence and other atmospheric perturbations are virtually nonexistent and can be considered negligible.

## Probing complex media through the propagation properties of vortex light

Vortex beams provide critical insights into light-matter interactions, revealing valuable information about the dynamics of complex media. Their unique transmission properties make them particularly effective for remote sensing in various environments, allowing the detection of subtle variations in parameters such as fluid velocity^105–108^, temperature, humidity, density, refractive index^109^, turbulence^110^, and scattering of suspended particles. Examining the transmission properties of vortex beams through complex media offers technical support for the classification and inversion of these media parameters. Furthermore, integrating deep learning algorithms significantly enhances the analytical capabilities of vortex beams. These algorithms assist in interpreting complex data patterns and correlations, thereby improving both the accuracy and scope of environmental assessments and monitoring. This synergy not only refines measurement precision but also expands the range of potential applications across various fields.

**Fig. 8:**
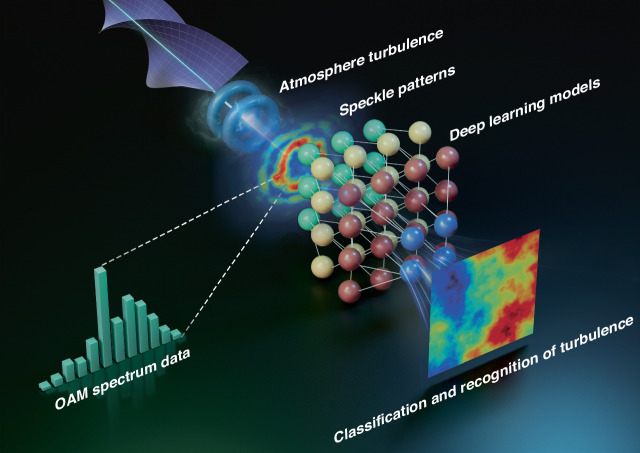
Leveraging vortex beam propagation characteristics for enhanced environmental monitoring. As vortex beams propagate through the atmosphere, turbulence disrupts their intensity distribution and modifies their spiral phase structure, leading to the formation of speckle patterns and the dispersion of the OAM spectrum. Analyzing these effects provides valuable insights into atmospheric properties, aiding in the retrieval of key environmental parameters. Moreover, combining two-dimensional speckle patterns with one-dimensional OAM spectrum data enables deep learning models to accurately classify and reconstruct turbulent atmospheric conditions

In atmospheric environmental monitoring, vortex beams are recognized as effective tools for probing turbulent parameters due to their high sensitivity to minute changes in the refractive index of the atmospheric environment, owing to their unique intensity and phase structure and their carrying of OAM, as shown in Fig. 8. During transmission through the atmosphere, their intensity distribution gradually breaks up and forms speckle patterns due to atmospheric disturbances^110^. These speckle patterns contain a wealth of atmospheric information and are crucial for extracting turbulent characteristic parameters, including scale and strength. Leveraging neural network models to process speckle patterns, particularly for correcting turbulent aberrations in perturbed vortex beams and identifying their carried OAM modes in the atmosphere, has significantly mitigated the adverse effects of turbulence^111^.

**Fig. 9:**
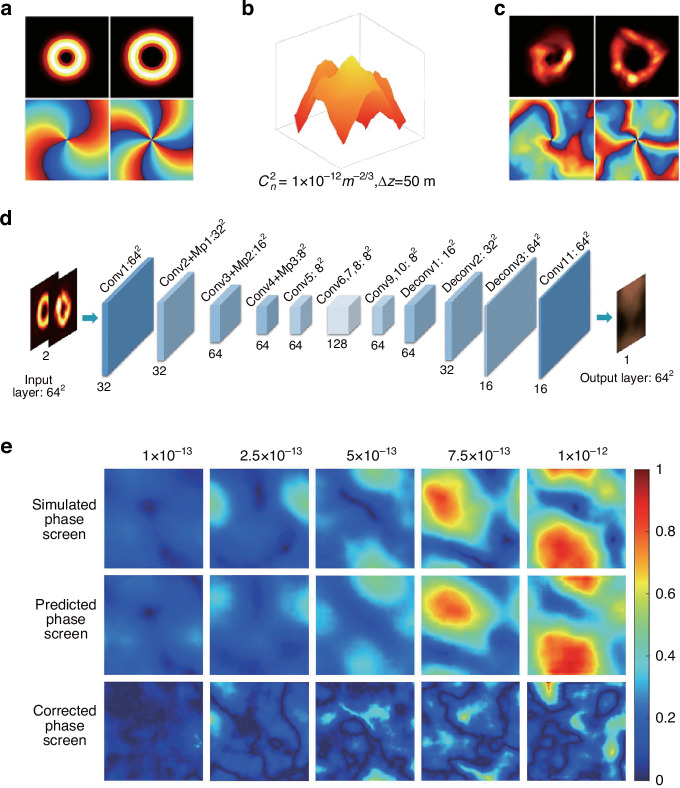
Atmospheric parameter inversion and correction based on vortex beam speckle patterns. **a**–**c** The influence of atmospheric turbulence (AT) on vortex beams with different OAM mode numbers (*l* = 2, 4) at *Δz* = 50 meters. **a** Intensity and phase distributions of undisturbed light beams. **b** Simulated AT equivalent phase screen. **c** Intensity and phase distributions of the vortex beams after disturbance. **d** Schematic diagram of the CNN framework used for AT compensation. Conv convolutional layer, Mp Max-pooling layer, Deconv deconvolution layer. **e** The simulated, predicted, and compensated phase screens under different ATs. Adapted with permission from ref. ^114^

An innovative method for adaptive atmospheric aberration correction employing convolutional neural networks (CNN) has been proposed, which derives Zernike polynomial coefficients by examining speckle patterns of vortex beams under various atmospheric conditions and provides a novel approach to compensate for turbulent aberration^112^. Additionally, an advanced deep CNN model has been developed to compensate for multiple distorted vortex beams simultaneously, correlate speckle patterns of vortex beams with atmospheric turbulence aberrations, and significantly improve the purity of OAM modes^113^. The strategy for aberration corrections was refined by analyzing the speckle patterns that arise when vortex beams pass through atmospheric turbulence^114^, allowing for the determination of turbulence characteristics as shown in Fig. 9. The CNN-based aberration correction model effectively mitigates atmospheric turbulence, significantly enhancing the transmission quality of phase distribution and the purity of OAM modes in vector vortex beams^115^.

The effective utilization of artificial neural network technology has significantly improved the recognition of 16 overlapping OAM modes transmitted through atmospheric turbulence, achieving an accuracy rate of 98.3%^116^. In addition, the robust classification capabilities of fully connected neural networks are evident in the detection of high-order multimode vortex beams, maintaining an accuracy rate of over 74% on 110 multimode vortex beam types, even under low signal-to-noise ratio conditions^117^. The integration of CNN algorithms has been crucial for the further development of adaptive demodulators, enabling the accurate recognition and interpretation of vortex beams with different OAM modes without the need for additional hardware^118^. Moreover, an optical feedback network efficiently and dynamically regulates the distribution of OAM modes under different atmospheric turbulence conditions by training CNN models to analyze speckle patterns of perturbed vortex beams and subsequently adapting them to match the desired target patterns^119^. Furthermore, a strategy employing deep feedforward neural networks has been presented that exhibits unprecedented accuracy in detecting the OAM modes of mixed-state vortex beams, with a recognition accuracy of more than 99%, even in atmospheric turbulence of up to 1000 meters^120^. The ability to recognize OAM modes in the midst of atmospheric turbulence was significantly improved by integrating a combination of post-interference and original intensity patterns into a CNN framework^121^. By adopting a CNN model to analyze the speckle patterns of disturbed vortex beams, information on atmospheric turbulence was successfully extracted, resulting in the correction of turbulent aberration and identification of OAM modes. The integration of these patterns with OAM spectrum data has significantly enhanced the accuracy and efficiency of detecting OAM modes in atmospheric turbulence^122^.

Significant progress has been made in the classification of atmospheric turbulence by analyzing speckle patterns of vortex beams during atmospheric transmission, enabling systematic categorization and reconstruction of six primary levels of atmospheric turbulence^123^. The use of the LeNet-5 neural network has proven to be highly effective in accurately classifying and reconstructing different types of turbulence encountered in atmospheric transmission studies, covering 5 different turbulence intensities^124^. Furthermore, the support vector machine (SVM) has proven effective in classifying and retrieving atmospheric environmental features by utilizing characteristics such as scintillation index, beam width, and beam wander across atmospheric transmission to efficiently identify the OAM modes of vortex beams^125^. Moreover, the application of the ResNet-101 model in a transfer learning framework has yielded remarkable results in accurately identifying both the OAM mode and transmission distance of vortex beams, even under varying turbulent conditions^126^.

**Fig. 10:**
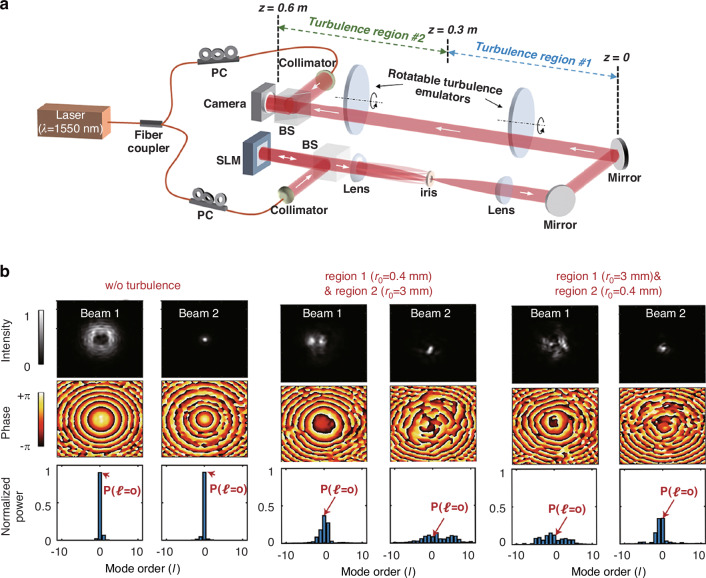
Experimental results for beam width measurement and modal coupling in two distinct turbulence regions. **a** The experimental setup utilizes two longitudinally structured beams, each with narrow beam widths at 0 < *z* < 0.3 m and 0.3 m < *z* < 0.6 m, respectively. **b** Experimentally measured intensity and phase profiles, along with the normalized modal spectra of Beams 1 and 2, for two different turbulence cases where regions 1 and 2 have different *r*_0_ values. Adapted with permission from ref. ^127^

On the other hand, atmospheric turbulence can also distort the helical phase structure of vortex beams, leading to crosstalk among the OAM modes and dispersing the OAM spectrum, as shown in Fig. 8. Analyzing the OAM spectrum of a perturbed vortex beam can also reveal the atmospheric transmission properties of vortex beams and provides a unique opportunity to infer atmospheric turbulence parameters^110^. However, the potential of this approach has not yet been fully explored. The detection of turbulence involves the transmission of longitudinally structured light beams with multiple Bessel-Gaussian modes, leading to variations in beam width and facilitating the coupling of OAM modes, depending on the distance traveled and the turbulence encountered, as shown in Fig. 10. Despite the turbulence structure constant fluctuations of up to 30 dB over a distance of 10 kilometers, this method allows for accurate measurement of the turbulence-induced OAM mode power coupling at the receiver, enabling derivation of the turbulence intensity distribution along the propagation path with consistent detection accuracy^127^.

The use of SVM models trained on OAM spectrum data derived from perturbed vortex beams allows both the Reynolds number and the Fried parameter in atmospheric turbulence to be determined independently while evaluating variations in experimental parameters such as temperature and wind speed. This approach, using OAM spectrum probes to measure temperature and wind speed, offers significant performance advantages over conventional techniques based on scintillation index analysis^128^. Speckle patterns of vortex beams during atmospheric transmission, along with their dispersed OAM spectrum, contain a wealth of information about atmospheric characteristics, with speckle patterns revealing local atmospheric details and the OAM spectrum revealing global atmospheric structure. Previous machine learning methods for assessing turbulence primarily focused on either distorted intensity or phase information (such as OAM) separately. However, by merging these two datasets or integrating additional characteristic data from disturbed vortex beams, a multiscale feature fusion approach can significantly improve the accuracy, precision, and training speed for atmospheric parameters classifying and inversion.

## Quantum sensing with the OAM modes of vortex light

In classical optical metrology, the central limit theorem indicates that the statistical error associated with a measurement outcome can be significantly reduced by an amount proportional to *n*^−1/2^, simply by repeating the measurement *n* times and averaging the results. In contrast, the emerging field of quantum metrology surpasses the limitations of classical metrology by reducing the error proportional to *n*^−1^^129^. This not only reduces the error but also pushes beyond the standard quantum limit, previously considered an insurmountable benchmark. This advancement is enabled by the strategic use of unique quantum resources, particularly entanglement^130^. NOON states, defined as a superposition of *N* photons in one path and none (0) in the other, have become fundamental in quantum metrology^131^. However, these states are highly sensitive to loss and noise, which can add or subtract photons, altering the values of *N* and 0, and thereby compromising the measurement accuracy.

**Fig. 11:**
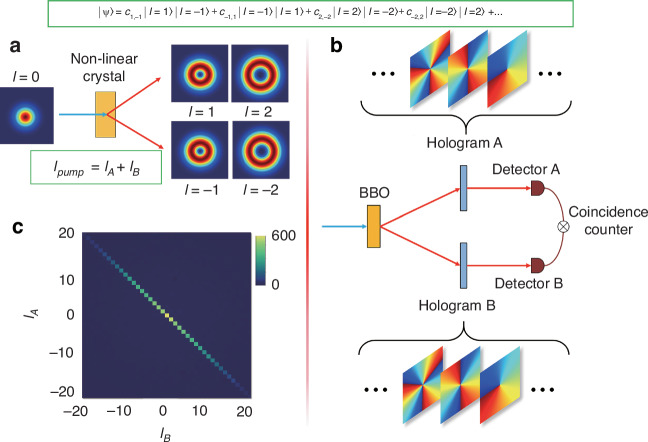
OAM entanglement. **a** OAM entanglement is generated via Spontaneous Parametric Downconversion (SPDC) in a nonlinear crystal, where the OAM of the two SPDC photons, adding to that of the pump, *l*_pump_ = *l*_*A*_ + *l*_*B*_, e.g., if the pump has zero OAM, then if *l*_*A*_ = 1 corresponds to *l*_*B*_ = −1, and so on, as shown in the insets. This process results in an infinite superposition state, represented by $\left\vert {\rm{\Psi }}\right\rangle$. **b** Measurement process involves a quantum version of the previously discussed modal decomposition method, typically employing holograms encoded onto SLMs, with the resulting light coupled into single-mode fibers, and the outcomes are then measured in coincidence using a coincidence counter. For OAM measurements, the holograms correspond to the standard azimuthal phases, as depicted in the insets. **c** Experimental validation confirms that OAM naturally forms a Schmidt basis, thereby verifying the conservation of OAM at the single-photon level

Only two decades ago, OAM was conclusively demonstrated as a basis for entanglement^132^. Below is a brief summary of OAM as entangled states: Classical OAM states are created by modulating the light either internally or externally to the source, and subsequently measured. In this context, the creation and detection steps are distinct. In quantum entangled OAM, the OAM is typically post-selected by measurement, following the principle in quantum mechanics that “you get what you measure." A typical OAM entanglement experiment, conceptually illustrated in Fig. 11a, involves passing a high-energy photon through a nonlinear crystal, where it undergoes spontaneous parametric down-conversion (SPDC), yielding two lower-energy photons as output. These photons are entangled in position and linear momentum, but the conservation of OAM at the single-photon level implies that the quantum state can also be expressed on an OAM basis. For instance, if the pump photon has zero OAM, then the OAM of photon A and photon B must likewise add to zero, producing the quantum state: $\left\vert \psi \right\rangle ={c}_{0}{\left\vert 0\right\rangle }_{A}{\left\vert 0\right\rangle }_{B}+{c}_{1}{\left\vert 1\right\rangle }_{A}{\left\vert -1\right\rangle }_{B}+{c}_{1}{\left\vert -1\right\rangle }_{A}{\left\vert 1\right\rangle }_{B}+{c}_{2}{\left\vert 2\right\rangle }_{A}{\left\vert -2\right\rangle }_{B}+\ldots$.

The measurement of each photon is performed by running the creation steps in reverse and coupling the output to a single-mode fiber, which acts as an OAM-sensitive detector for single photons^133^. The outcomes are then measured in coincidence, revealing the quantum nature of the correlations, as illustrated in Fig. 11b. These final states can be utilized for various high-dimensional quantum protocols with structured photons^134^, including high-dimensional communication^135^. Additionally, we observe a high-dimensional state that forms a natural Schmidt basis, with an example experimental outcome shown in Fig. 11c.

**Fig. 12:**
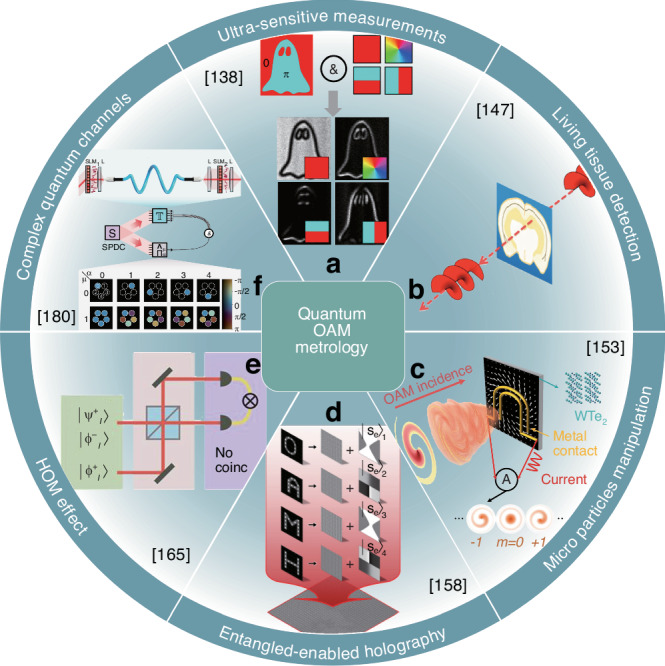
Advanced applications of quantum OAM metrology. **a** Enhanced precision in physical quantity measurements through OAM quantum states, achieving superior accuracy and sensitivity. **b** Quantum OAM metrology for advanced detection and imaging of living tissues. **c** Interaction of OAM beams with particles, enabling precise control and manipulation at microscopic scales. **d** Generation of high-resolution holography using entangled photon pairs, enhancing imaging techniques in terms of depth and clarity. **e** Utilization of the HOM effect for precise quantum state discrimination, advancing quantum computing and information processing capabilities. **f** Detailed study and characterization of complex quantum communication channels, which enhances the understanding of quantum information transfer mechanisms. References are given in the insets

This finding has opened numerous opportunities, allowing researchers to access vast, high-dimensional Hilbert spaces, facilitated by the concept’s theoretically unbounded alphabet ranging from −*∞* to *∞*. This unique resource has garnered significant interest, as evidenced by the growing body of literature on the subject^133,134^. In this section, we will explore recent advancements that have transformed the degree of freedom in quantum states of light, expanding beyond conventional photon number, polarization, position, and paths to include spatial modes and OAM. Additionally, we will highlight the benefits these innovative approaches bring to the field of metrology, enhancing the precision and accuracy of measurements, as illustrated in Fig. 12.

A quantum version of digital spiral imaging, a technique that allows for the non-local analysis of an object by its OAM spectrum, has been developed. This innovative method relies on the creation of two OAM-entangled photons. One of these photons is directed towards the unknown object, while the other is sent to the usual modal decomposition. Importantly, the OAM spectrum is measured on the photon that did not interact with the object. In an early version of this experiment, the object arm incorporated fractional OAM phase masks^136^. However, the experiment has since been refined and is now executed with arbitrary spatially resolved phases^137^. This technique has even been applied to physical angular slits, enabling researchers to discern the slit width and resolve edges in quantum objects^138^. Remarkably, there is no immediate resolution enhancement derived from the entanglement itself, other than the unique capability for a non-local interaction. However, the rotation of physical and phase objects could potentially be detected by combining OAM spectrum analysis with its rotational frequency shift^139^.

Despite certain potential limitations, OAM presents itself as a promising tool for ultra-sensitive measurements of rotations^140^, as illustrated in Fig. 12a. To elucidate this potential, consider a superposition state of the form $\left\vert \psi \right\rangle =\left\vert l\right\rangle +\left\vert -l\right\rangle$ that undergoes a rotation by *θ* for each mode of *l*. The final state will be $\left\vert \psi \right\rangle =\exp ({\rm{i}}l\theta )\left\vert l\right\rangle +\exp (-{\rm{i}}l\theta )\left\vert -l\right\rangle$, resulting in a rotational enhancement $\Delta \theta =1/(l\sqrt{\nu })$, where *ν* represents the number of single-photon probes. OAM states exhibit NOON-like interference fringes and achieve sensitivity improvements by a factor of *l*. This approach has been successfully demonstrated using path-entangled OAM photons to measure angular displacements with enhanced resolution and sensitivity, exceeding the classical limit^141^. Moreover, this improvement factor of *l* has been corroborated up to remarkably high OAM values^142^, introducing the concept of photonic gears, which amplify rotation sensitivity based on OAM^143^. A key feature of these pioneering works is the necessity for the OAM axis and rotation axis to be ideally aligned. However, this requirement can be overcome by constructing OAM in Majorana constellations, enabling quantum-enhanced rotation measurement even when the rotation axis is unknown^144^. This methodology has applications in estimating the three parameters of a general rotation^145^.

Historically, it was generally assumed that OAM would not provide any direct benefits in the detection of chiral structures. This assumption was based on the fact that no immediate mechanism could be identified^146^. However, this perspective has since shifted and evolved. New studies have revealed the critical importance of quadrupole fields, and, in recent times, OAM photons have even been successfully utilized to probe living tissue^147^, as depicted in Fig. 12b. The broader comprehension of light-matter interactions with OAM has prompted numerous advancements when employing classical light^148^. However, these advancements haven’t been equally evident at the quantum level. In a significant, yet largely overlooked breakthrough, it was demonstrated that multiwavelength polarization-entangled photon pairs could effectively measure the optical activity of chiral molecules, outperforming conventional classical methods^149^. However, despite this progress, no advancements have yet been reported in chirality detection using quantum OAM states. This absence of progress is likely attributable to the fact that the required quadrupole excitations are incredibly weak. Consequently, developing methods for chirality detection using quantum OAM states remains an unresolved challenge, presenting an opportunity for further exploration by the scientific community^150^.

Despite the scarcity of experiments, there have been a multitude of quantum-inspired advances, with the manipulation of atoms using OAM being substantial and well-reviewed to date^151^. A seminal advance in light-matter interaction with OAM was the demonstration of OAM transfer to a bound electron^152^. This work involved an ion-trapping experiment that revealed a previously unknown selection rule in atomic physics. The experiment showed that OAM could be transferred to the valence electron of a single trapped ion. This intriguing observation can only be reasonably explained by the fact that the electron was able to absorb two quanta of angular momentum from a solitary photon, one from the photon’s polarization and the other from its spatial structure (OAM). The study also addressed a previous impediment: how can OAM be conveyed on the axis if there is an absence of light there? The authors elucidated that it is the gradient of the field, rather than the field itself, that holds significance. This work has led to several crucial advancements in the manipulation of electronic states using OAM light, as shown in Fig. 12c. These developments include the orbital photogalvanic effect^153^ for circulating currents based on the OAM of light (phase driven), imprinting a vectorial current on matter through coherent control of two quantum pathways during the excitation process^154,155^, and imprinting OAM onto a propagating electron matter wave, thereby inducing an OAM-dependent dichroic photoelectric effect^156^. All these advances are collectively nurturing a novel form of single-photon electron spectroscopy and might potentially be applied in light-controlled chemistry utilizing OAM^157^.

A novel and promising application is emerging, which is in the remote analysis and information sharing of samples through the use of entangled-enabled holography^158^. This technique, illustrated in Fig. 12d, exhibits remarkable resilience to classical noise, rendering it an attractive choice for various applications. While the primary focus of this recent study was to showcase the concept, it quickly became apparent that this method could be seamlessly applied to all the traditional application areas of holography. These areas range from medical imaging to security purposes and manufacturing processes. What sets entangled-enabled holography apart is that it adds a non-local flavor to these applications. This implies that the object under examination and the hologram can now be located at different, distant locations, thereby opening up novel possibilities for collaboration and data sharing on a global scale.

Another quantum resource that has traditionally found application in metrology is the Hong-Ou-Mandel (HOM) effect, depicted in Fig. 12e. Originally introduced as a means to measure extremely short time intervals, with a resolution down to 1 fs^159^. This capability was subsequently extended to attosecond resolution^160^, allowing for even more precise temporal measurements. Additionally, the HOM effect has been applied to the realm of quantum microscopy^161^, enabling researchers to study phenomena at an even smaller scale. In the context of spatial modes, the HOM effect has been utilized as just such a ’stop-watch’ to probe OAM photons in flight, detecting small phase differences that may arise due to their unique structure^162–164^. Moreover, the HOM is also crucial in OAM quantum state engineering^165,166^, where it is used for measuring^167^ and filtering^168^ quantum states.

One of the most compelling exploration topics is the utilization of quantum OAM light to probe and transverse complex quantum channels, as illustrated in Fig. 12f. The striking isomorphism between the quantum state and quantum channel imparts a profound understanding: a measurement executed on one entity is, in essence, tantamount to a measurement undertaken on the other. This insight has also paved the way for the enlightening realization that certain classical OAM states bear a striking resemblance in behavior, albeit not encompassing all properties to their quantum counterparts, especially when their trajectories are charted within the intricate terrain of a complex channel^169,170^. Such parallels have been skillfully harnessed, facilitating the innovative use of classical light to enrich quantum processes, or, conversely, employing a quantum toolkit to meticulously dissect classical states, thereby spearheading the emergence of the novel discipline of classical entanglement^171,172^.

Quantum states of OAM, as one might expect, have been extensively deployed for the purpose of quantum communication^173,174^, but have also been aptly utilized for the task of probing and conveying entanglement across complex channels^175,176^. In such scenarios, the very act of entanglement decay can serve as a direct measure of the channel, and, when set against the backdrop of high-dimensional OAM, enables a parallel interrogation of the system^177,178^. Such intricate systems lend themselves naturally to machine learning and artificial intelligence so that the outcoming state can either be meticulously corrected or, alternatively, employed as a discerning probe of the intricate environment it has traversed^179^. Conversely, the medium’s inherent complexity could be strategically harnessed as an invaluable tool for the precision engineering of the desired quantum state and circuit^180^.

## Conclusion

The review highlights significant advancements in optical metrology using vortex beams, characterized by their unique spiral phase structure and carried OAM. Vortex beams benefit optical metrology applications, enabling highly sensitive detection of chiral interactions between light and matter, as well as precise 3D motion detection via linear and RDE. They effectively probe complex media, with applications in environmental monitoring, deep tissue imaging, and noisy channel communication. By combining complexity and artificial intelligence, these techniques can be further refined. Vortex beam metrology has revolutionary applications and holds great promise for driving advancements in optical metrology.
